# Differential Gene Expression in Tamoxifen-Resistant Breast Cancer Cells Revealed by a New Analytical Model of RNA-Seq Data

**DOI:** 10.1371/journal.pone.0041333

**Published:** 2012-07-23

**Authors:** Kathryn J. Huber-Keener, Xiuping Liu, Zhong Wang, Yaqun Wang, Willard Freeman, Song Wu, Maricarmen D. Planas-Silva, Xingcong Ren, Yan Cheng, Yi Zhang, Kent Vrana, Chang-Gong Liu, Jin-Ming Yang, Rongling Wu

**Affiliations:** 1 Department of Pharmacology, The Penn State Cancer Institute, The Pennsylvania State University College of Medicine, Milton S. Hershey Medical Center, Hershey, Pennsylvania, United States of America; 2 The Center for Statistical Genetics, The Pennsylvania State University College of Medicine and Milton S. Hershey Medical Center, Hershey, Pennsylvania, United States of America; 3 Department of Experimental Therapeutics, MD Anderson Cancer Center, Houston, Texas, United States of America; 4 Department of Applied Mathematics and Statistics, State University of New York, Stony Brook, New York, United States of America; Harvard School of Public Health, United States of America

## Abstract

Resistance to tamoxifen (Tam), a widely used antagonist of the estrogen receptor (ER), is a common obstacle to successful breast cancer treatment. While adjuvant therapy with Tam has been shown to significantly decrease the rate of disease recurrence and mortality, recurrent disease occurs in one third of patients treated with Tam within 5 years of therapy. A better understanding of gene expression alterations associated with Tam resistance will facilitate circumventing this problem. Using a next generation sequencing approach and a new bioinformatics model, we compared the transcriptomes of Tam-sensitive and Tam-resistant breast cancer cells for identification of genes involved in the development of Tam resistance. We identified differential expression of 1215 mRNA and 513 small RNA transcripts clustered into ERα functions, cell cycle regulation, transcription/translation, and mitochondrial dysfunction. The extent of alterations found at multiple levels of gene regulation highlights the ability of the Tam-resistant cells to modulate global gene expression. Alterations of small nucleolar RNA, oxidative phosphorylation, and proliferation processes in Tam-resistant cells present areas for diagnostic and therapeutic tool development for combating resistance to this anti-estrogen agent.

## Introduction

Tamoxifen (Tam) is commonly used as an adjuvant hormonal therapy for patients with breast cancer. This selective estrogen receptor modulator (SERM) blocks the effects of estrogen in breast cancer cells by competitively interacting with the estrogen receptor (ER), thus preventing ER-mediated transcription through estrogen response elements of various genes. While conventionally used in ER-positive tumors, which comprise approximately 70% of breast cancers [1], in recent years Tam has also been used to successfully treat some ER-negative breast tumors [2]. Even so, the benefits of hormonal therapy have often been limited by resistance to this drug. Approximately one-third of early-stage breast cancer patients will become resistant to Tam over the 5-year treatment period [3], making resistance to Tam treatment one of the major obstacles to the successful treatment of breast cancer. Although studies have already revealed several mechanisms of Tam resistance, including increased metabolism of Tam [4], loss or alterations of ERα and ERβ expression [5], [6], [7], estrogen hypersensitivity [8], altered expression of co-regulators [9], and microRNA (miRNA) interference [10], many of these investigations focused on individual types of mechanisms and lacked global analysis of gene expression and signaling pathway alterations for association with the development of Tam resistance. While global microarray studies have been performed [11], [12], some were limited to a chosen set of genes, while others were genome-wide studies that still did not include small RNA analysis and focused instead on the protein-coding genome [13], [14]. In order to improve the effectiveness of Tam therapy, a more comprehensive understanding of the molecular mechanisms and pathways determining Tam sensitivity would help overcome this clinical problem.

In the current study, next generation sequencing (NGS) technology was used to identify the genes and pathways potentially involved in Tam resistance through a global analysis of the transcriptomes in Tam-sensitive (TamS) and Tam-resistant (TamR) breast cancer cells. NGS, or deep sequencing, offers a powerful platform for characterization of altered gene expression, as it allows for a more unbiased exploration of all areas of the genome and transcriptome. RNA-Seq can overcome microarray-associated problems with cross hybridization of similar sequences and allows for single nucleotide resolution, as well as reducing under-representation or the omission of low abundance sequences [15]. Although one study has been recently published using NGS to explore tamoxifen resistance [16], this investigation used deep sequencing to identify hits from an shRNA screening library.. While it is recognized that prior biological knowledge can be important in developing some biologically relevant clustering models, new relationships between molecules can be missed by using such a technique. Thus, we present an alternative analytical method.

As the RNA-Seq field is relatively new, analysis models must be tested and compared for their ability to accurately analyze genomic data. Traditional approaches for pattern identification, such as hierarchical clustering or other partitioning methods, are based on cluster analysis for differential gene expression under one specific condition or treatment [17], without considering the mechanisms behind differential expression across environments. These approaches can cluster genes into different groups according to their known functions, but are not able to catalogue genes based on the patterns of how different genes respond to different environmental signals. The difference in expression of the same gene between environments, called phenotypic plasticity, plays an important role in the adaptation of organisms or cells to environmental changes [18], [19]. Therefore, we developed an algorithmic model for clustering genes based on environment-dependent differences and ratios by incorporating these measures into a mixture model framework, in which an optimal number of gene clusters can be estimated and the patterns of gene expression plasticity tested. Because of the integration of intrinsic environment-dependent plasticity, results from our model are biologically more relevant than those from traditional clustering approaches using a single environment, which rely on known functional similarities or a predetermined number of gene clusters.

Using this new method, we found that large global changes occur in TamR cells, with differential expression of many genes involved in transcriptional/translational control as well as cell cycle and mitochondrial dysfunction. Through clustering, we identified patterns of differential expression in response to differences between TamS and TamR cells, with similar functions often clustered together in expression. Through our approach, 1215 mRNA and 513 small RNA (smRNA) transcripts were identified as significantly differentially-expressed, indicating that resistance to Tam is multi-faceted, derived from global changes in gene expression, and involves multiple pathways.

## Results and Discussion

The objective of our study was to overcome the limitations of previous studies by developing a comprehensive analysis of the transcriptome changes involved in Tam resistance in breast cancer using the NGS method. NGS allows for unbiased analysis and exploration of all possible cellular molecules and pathways. Tam resistance is a complex problem, and the field would benefit tremendously from studies examining global changes with NGS, which have not been previously explored.

### Validation and comparison of gene expression levels between Tam-sensitive and Tam-resistant breast cancer cells

**Figure 1:**
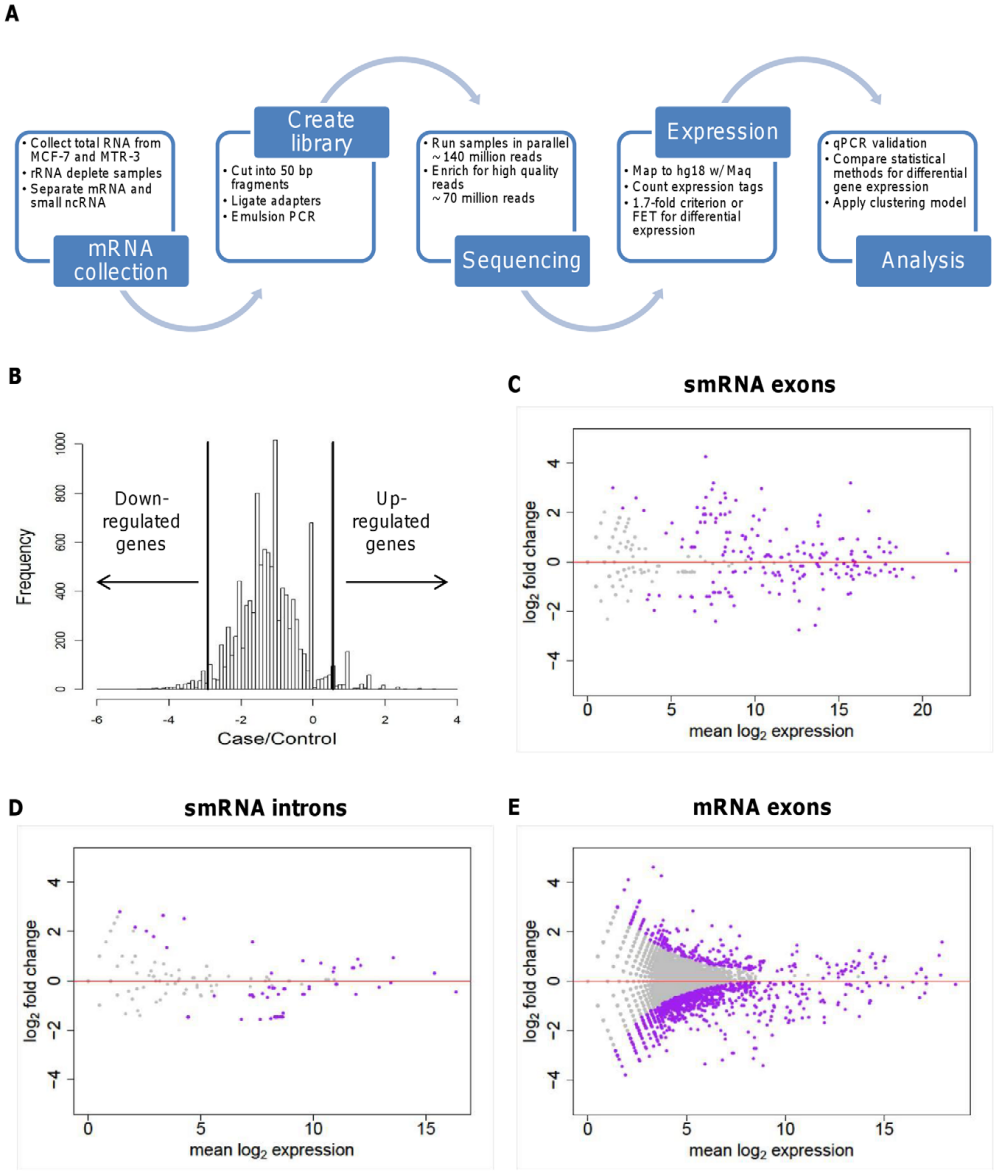
NGS identification and comparison of differentially-expressed genes in TamR cells by the Fisher's exact test. (**A**) Total RNA from human breast cancer cell lines MCF-7 (TamS) and MTR-3 (TamR) were collected and subjected to the next generation sequencing process. (**B**) Gene expression followed a Poisson distribution with significantly differentially-expressed genes two standard deviations from the mean in the traditional method. The new method used the FET significance test. The change of the normalized smRNA exon reads (**C**) and intron reads (**D**), and exon reads for mRNA genes (**E**) from TamS to TamR cells is plotted against the mean expression between these two types of cells for the new method. Purple dots represent significantly expressed genes as determined by FET; gray dots represent genes with similar expression. The red horizontal line at zero provides visualization for the signs of differential expression.

In order to reveal the potential genes and mechanisms involved in resistance to Tam, we used a NGS approach with ABI SOLiD3 technology as a means of examining and comparing the transcriptomes of TamS and TamR breast cancer cell lines. These cell lines were previously characterized for tamoxifen resistance [20], [21], which was confirmed before sequencing. Experimental procedures are summarized in Figure 1A. A total of 71,250,509 and 69,005,180 reads, for TamS and TamR cells respectively, were sequenced. Gene expression of parental MCF-7 (TamS) cells was used as a baseline for up- or down-regulation of expression in TamR cells. Gene expression data by RNA-Seq are generally thought to follow a Poisson distribution . [22]. To check whether our data are Poisson-distributed, we calculated chi-square goodness of fit test statistics for read counts observed in TamS and TamR cell lines, respectively. The calculated test statistics by assuming the Poisson distribution are smaller than critical thresholds, suggesting that these RNA reads obey a Poisson distribution (*P*>0.90). Based on this two-standard deviation criterion of mRNA expression which indeed followed a Poisson distribution (Fig. 1B), we found that 667 mRNAs were significantly differentially-expressed between the TamS and TamR cell lines.

**Figure 2:**
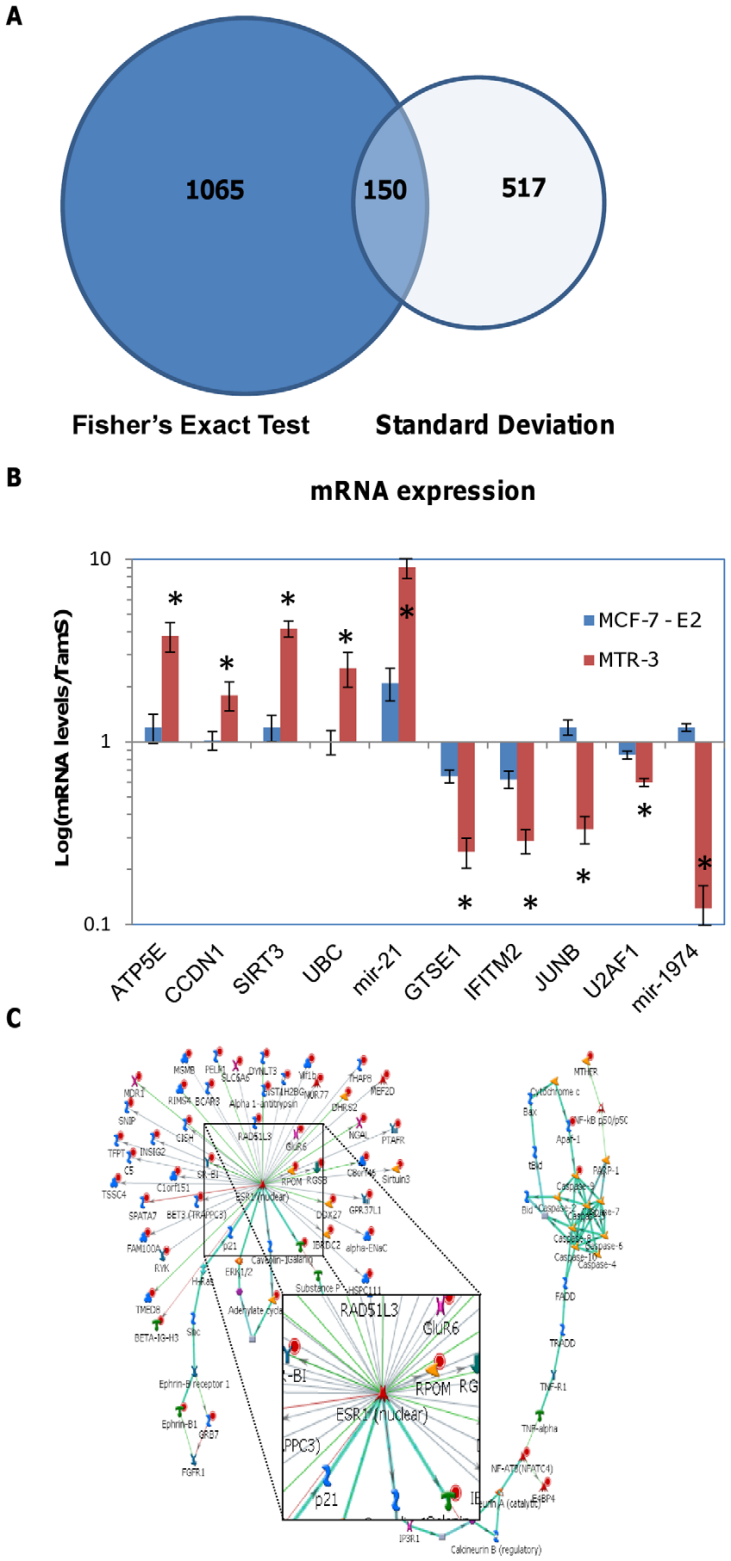
Comparison and validation of differentially-regulated genes by the two significance methods. (**A**) Venn diagram of overlap of significant genes found by simple calculation of two standard deviations or the Fisher's exact test. (**B**) Validation of mRNA levels of selected genes found by NGS was performed on MCF-7 (TamS), estrogen independent TamS cells (MCF-7-E2), and MTR-3 (TamR) cells by qRT-PCR. The log ratio of MCF-7-E2 or TamR to TamS gene expression is shown to indicate up- or down- regulation. GAPDH was used as a control. Each point represents mean ± S.D. of triplicate determinations; results shown are the representative of three identical experiments. *p<0.05; *t*-test. (**C**) GeneGO (Thomson Reuters) network analysis of the most significant networks dysregulated in TamR cells. Red circles with a red dot in the middle next to the proteins indicate up-regulation in TamR cells. The different shapes indicate different classes of proteins. Green lines indicate activation while red lines indicate inhibition; gray lines are unspecified interactions.

To better analyze and categorize the transcriptome differences associated with Tam resistance, including analysis of smRNA, we used the Fisher's Exact Test (FET), in which significance was assessed with the normalized data by FPKM (fragments per kilobase of exon per million fragments mapped). This allows for analysis of smRNA (which may map to unidentified genome regions with no recognized gene lengths) in addition to mRNA and more accurately deals with variation between different treatments or cell lines [23]. FET was therefore also used to analyze the significance of differential expression between the TamS and TamR cells for each gene, a method which has recently gained favor in microarray analysis [24]. Among a total of 7713 small RNAs, 513 display significant differences in exon reads (Fig. 1C) between the two cell types. For intron reads, 55 smRNAs were differentially-expressed (Fig. 1D). From a total of 23,561 mRNA genes, 1215 were differentially-expressed (870 up-regulated and 335 down-regulated) between the TamR and TamS cells (Fig. 1E). Interestingly, upon comparison of the mRNA expression, only 150 genes were found by both the “two-standard deviation” method and FET (Fig. 2A). Table 1 lists the most differentially-expressed genes found by both tests.

**Table 1:** Most differentially-expressed genes revealed by both significance tests.

Gene	Function	Fold-change	P-value
**Up-regulated Genes in TamR cells**			
ANKRD32	Ankyrin repeat domain: cell-cell adhesion and cell structure	12.25	0.015
ABHD10	Alpha-beta hydrolase	11.81	0.002
INTS12	Integrator complex subunit: associates with RNA polymerase II	9.19	2.00E-04
SIRT3	Sirtuin 3: deacetylase	9.19	.015
TATDN1	Putative deoxyribonuclease: alternative splicing	8.75	7.65E-05
UBC	Ubiquitin C: ubiquitination	8.75	0.008
CAV2	Caveolin-2: formation of caveolae	8.31	0.050
ATP5E	ATP synthase: oxidative phosphorylation	7.61	2.48E-81
HIST1H2BM	Histone 1: gene expression	7.44	4.31E-09
RAB27B	Ras oncogene: vesicular fusion and trafficking	7.00	0.003
**Down-regulated Genes in TamR cells**			
RPLP1	60S ribosomal protein: translation	−22.86	1.42E-12
SLC12A9	Solute carrier: membrane transport	−18.29	0.034
REEP6	Receptor accessory: cell surface receptor expression	−11.43	0.001
IFITM2	Interferon induced transmembrane protein: cell cycle arrest and apoptosis	−11.43	4.5E-05
NDUFS6	NADH dehydrogenase: oxidative phosphorylation	−9.14	.001
TSSC4	Tumor suppressing subtransferable:	−8.00	0.016
TMSB15B	Thymosin β: actin binding	−6.86	0.027
HIST1H3E	Histone 1: gene expression	−6.86	0.003
CSNK2A2	Casein kinase: PI3K and Wnt signaling	−6.10	0.016
ATP6V0E2	ATP synthase: oxidative phosphorylation	−5.94	0.007

For preliminary verification of differential expression between the TamS and TamR cell lines, we chose ten genes found by both statistical tests (five of which were up-regulated and five down-regulated in TamR cells) and compared their mRNA levels using quantitative RT-PCR. An additional treatment group of TamS cells grown in phenol red-free media, which acts as an estrogen mimic [25], was added to explore the effects of estrogen independence on the gene expression changes. Three replicates from cell culture experiments were prepared on three separate days that were distinct from those used for NGS. Figure 2B shows the mRNA levels of the selected genes as determined by qRT-PCR. The qRT-PCR confirmed the general up- or down-regulation of the genes. Quantitatively, the fold-changes observed were usually smaller in the qPCR analyses than the NGS by approximately 2-fold. The down-regulated genes in TamR cells, *GTSE1*,*IFITM2*, and *mir-1974* showed a 6-fold difference by NGS but only a 2 to 4-fold difference by qRT-PCR, while genes *CCDN1* and *U2AF1* showed a more moderate decrease of a 2-fold difference which was similar to their 1.7- difference found by NGS. Although all the down-regulated genes were more down-regulated in TamR cells than in TamS cells grown without estrogen, it was interesting that *JUNB* and *mir*-*1974* trended towards an up-regulation under estrogen independent conditions, suggesting a distinct mechanism for the emergence of tamoxifen resistance. TamR upregulated genes, *ATP5E*, *CCDN1*, *SIRT3*, *UBC*, and *mir-21*,a, showed a 7–9 fold difference by NGS but only a 2–4 fold difference by qRT-PCR. While *ATP5E*, *SIRT3*, and *mir-21* all had increased expression levels under estrogen independent conditions, all the up-regulated genes were increased further when the cells were tamoxifen resistant. Thus, while some of the validated genes have altered expression as they become estrogen independent, further alterations in expression appear to be necessary for the development of resistance to tamoxifen. An initial ontological exploration of both methods' sets of statistically significant genes indicated that genes related to *ESR1* (estrogen receptor alpha) comprised one of the most enriched pathways (Fig. 2C). This adds validation to the significance of our data set in comparison to previous studies [9], [26], [27].

### Phenotypic plasticity clustering analysis

**Figure 3:**
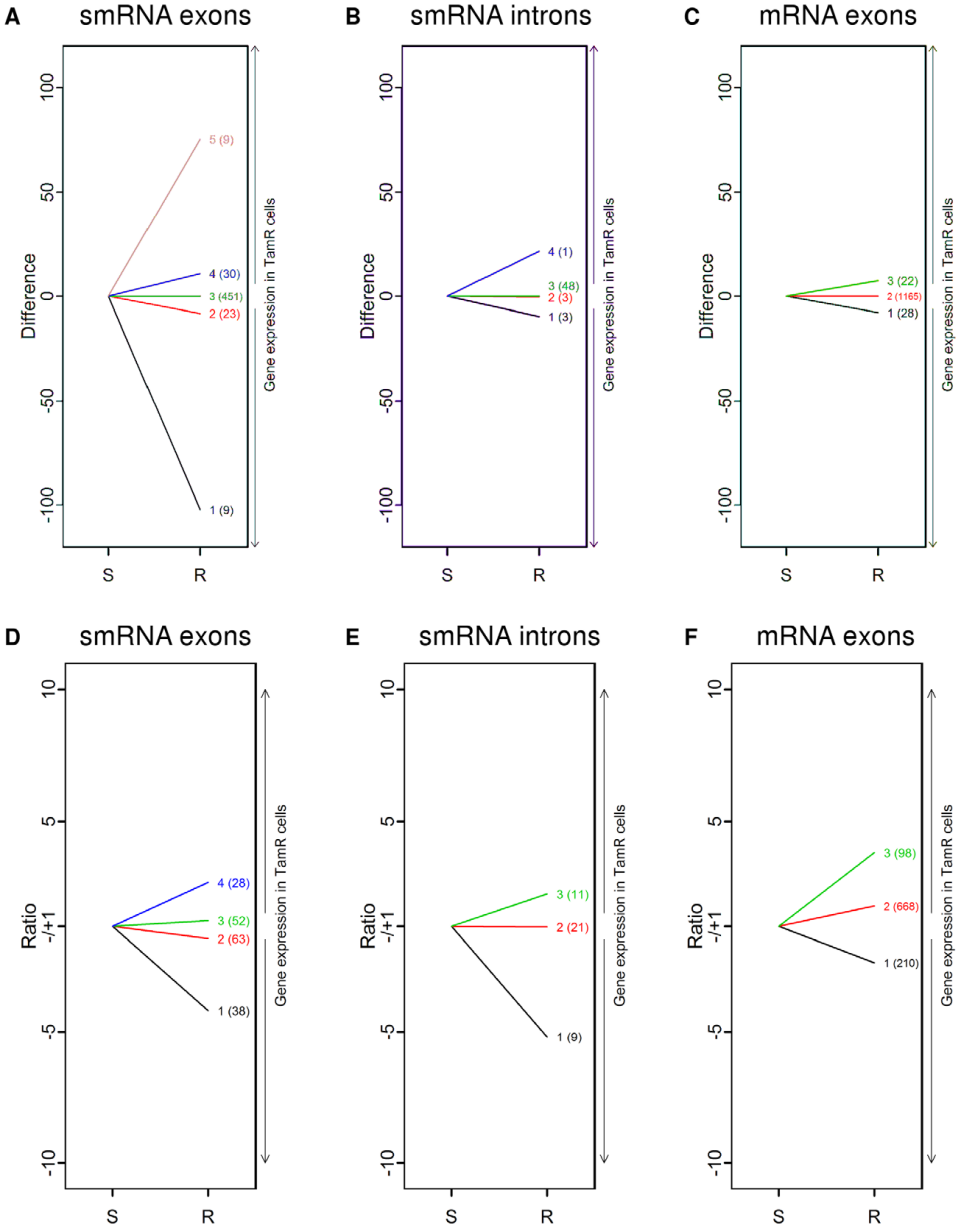
Clustering patterns of genes by absolute difference and ratio of expression. Clustering as determined by the difference model for smRNA exon reads (**A**) and intron reads (**B**), as well as mRNA genes for exon reads (**C**) in TamS (S) and TamR (R) cells. Clustering as determined by the ratio model for smRNA exon reads (**D**) and intron reads (**E**), as well as mRNA exon reads (**F**) in TamS (S) and TamR (R) cells. The number in parentheses corresponds to the number of genes in each cluster.

**Figure 4:**
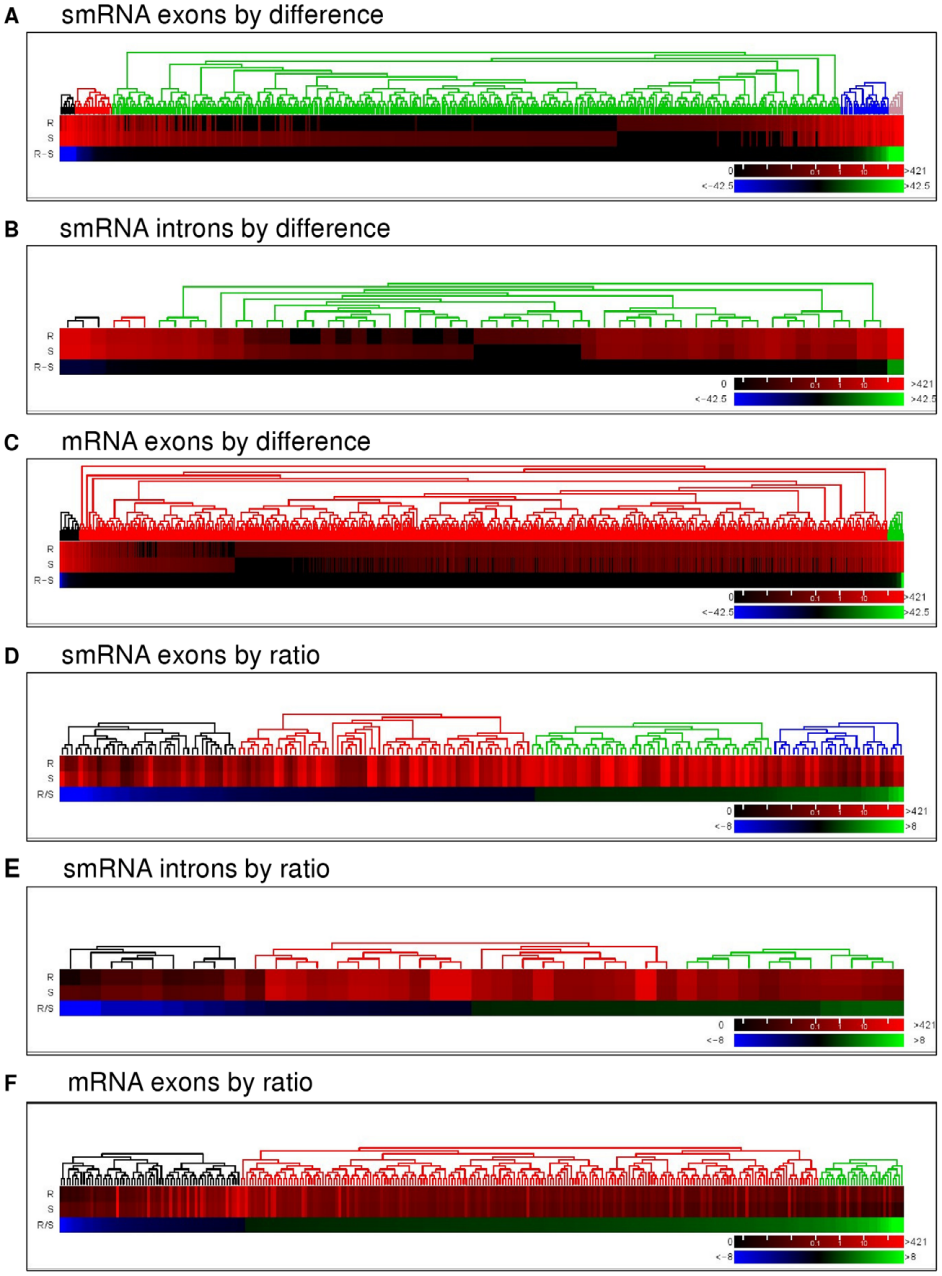
Heatmap comparison of differentially-expressed genes by clustering analysis. Heatmaps showing results of the clustering of small RNA exons (**A**) and introns (**B**), as well as mRNA exons (**C**) absolute difference gene expression (R-S) between TamS (S) and TamR (R). Heatmaps showing results of the clustering of small RNA exons (**D**) and introns (**E**), as well as mRNA exons (**F**) ratio gene expression (R/S) between TamS (S) and TamR (R). Gene expression levels are displayed for R and S on a log(absolute values) scale. (R-S) are absolute values while (R/S) values display a fold-change from R to S cells. Clustering groups are represented by different colors above the heatmaps. P-values were calculated using a χ-squared test.

Due to the large number of differentially-expressed genes, we next sought a method to categorize the genes based on their levels of differential expression to determine if any new patterns emerged. Because many traditional clustering methods create clusters based on known gene function similarities, they fail to recognize novel patterns of gene expression. Other methods that do not rely on gene function are usually limited because they force genes to fit into one of a predetermined number of gene clusters that can create false relationships between genes. To overcome these limitations, we developed difference and ratio models (see the Methods) that take into account phenotypic plasticity of gene expression and cluster the FET significant genes into different groups based on the pattern of differential expression between TamS and TamR cells (Figs. 3, 4). Phenotypic plasticity of gene expression can be measured as absolute differences or ratios of expression levels between environments or treatments. The difference model determines the assignment of genes to particular clusters based on absolute differences in gene expression levels from one environment (TamS) to the next (TamR), whereas the ratio model identifies expression patterns according to relative difference of gene expression. The optimal number of clusters is determined by a model selection criterion, such as the commonly used Akaike information criterion (AIC) [28] or Bayesian information criterion (BIC) [29]. in this study, both the AIC and BIC values under different numbers of clusters were calculated, with an optimal number of clusters corresponding to the minimum AIC value, which produced identical results for the optimal number of clusters. The two models for absolute difference and ratio of expression may also produce similar results, but meanwhile, they are complementary in identifying particular clusters. Detailed method and validation is unpublished as of yet.

The AIC and BIC criterioa calculated from the difference model both favor the choice of five clusters for the 513 exon genes and four clusters for 55 intron genes of the differentially-expressed small RNA genes. Figures 3A and 3B plot the patterns of absolute difference in smRNA gene expression in the TamS and TamR cells, showing marked differences in the pattern of differential expression. The majority of exon genes fall into Cluster 3 which represents low expression genes (Fig. 3A). It should be pointed out that for these weakly expressed clusters in both cell types, some sub-clusters may exist in terms of the relative difference which would be found with the ratio model. In general, the counts of intron reads are strikingly low compared with exon reads (Fig. 3B). Distinct patterns of absolute difference in gene expression can also be detected for total exon reads for mRNA. Introns were not included for mRNA analysis as they do not accurately portray the genes being expressed. Among 1215 significant mRNA genes, we detected three clusters based on both the AIC and BIC criteria (Fig. 3C), with the majority of genes falling into the low expression Cluster 2 with little absolute difference between the cell types. Overall, these results suggest that the difference model is effective for large differences in gene expression, but genes that have low expression could be inaccurately categorized as having no change between treatments.

The ratio model was better able to cluster genes together that had lower absolute expression but a high degree of difference in expression between TamS and TamR cells (Fig. 3D, E, F); up- and down-regulation is more evident in this format. In this model, fewer genes were clustered due to some genes only being expressed in one cell line. For smRNA gene expression, the model found four clusters for the exon-significant genes (Fig. 3D), while the absolute difference method found five (Fig. 3A). Intron-gene expression was clustered into three groups (Fig. 3E). For the differentially-expressed mRNA genes, the genes clustered into three groups again (Fig. 3F). As expected, the ratio model was better able to capture the nuances of the fold-changes than the absolute difference method, but the absolute difference method was superior at clustering genes simply by their absolute low or high levels of expression.

Taken together, this model was able to cluster differentially-expressed genes into groups with similar degrees of expression differences. With the model accurately taking into account the statistical ramifications of comparing across different environmental groups rather than just across multiple samples of the same treatment, our next goal was to determine if the genes within clusters have any significant known relationships to one another.

### Effects of Tam resistance on smRNA expression and clustering

In order to better understand how different types of smRNA were affected in TamR cells, we next examined the smRNA clusters. Based on clustering by absolute difference, almost all genes were designated to a single cluster (Cluster 3) in both the exon and intron analyses. These clusters were low expression genes that showed little difference between TamS and TamR cells when measured on an absolute expression scale (Fig. 3A–B). The majority of these differentially-expressed small RNA aligned to known small nucleolar RNA (snoRNA) genes as well as other non-coding RNA (ncRNA) regions. snoRNAs were both up- and down-regulated in TamR cells. This relatively new category of non-coding RNA was originally thought to be unimportant or to only have effects on the chemical modifications of other RNA molecules [30]. However, there is recent evidence showing that snoRNAs can act much in the same way as micro-RNA (miRNA), regulating gene expression [31]. Other ncRNA categories included those related to histone modification, small cajal nucleolar bodies, and vault RNA, with one notable exception: miRNA *mir-16-2* was found in Cluster 3. This miRNA normally stops E2F control of proliferation [32] and its down-regulation would allow proliferation to continue. smRNA exon Clusters 1, 2, and 5 contained only a few transcripts that were differentially-expressed, and all aligned sequences were mapped to snoRNA genes. The remaining group, Cluster 4, which contains moderately-expressed genes with little absolute difference but significant fold-change between TamS and TamR, did include one interesting transcript – *RMRP* (RNA component of mitochondrial processing endonuclease), a ncRNA that binds several proteins to create the endonuclease complex controlling mitochondrial transcription.

Comparison of the ratios of smRNA expression revealed much of the same alteration of snoRNA as well as other significant ncRNA. Most differentially-expressed smRNA that were not labeled as snoRNA were mapped to regions that were generally categorized as nonspecific ncRNA, open reading frames, and transcription regulation. However, there were some significant changes in miRNA. In Cluster 4, we found that known oncomir *mir-21* expression was increased in TamR cells by ∼5-fold, as was uncharacterized *mir-1259*. Up-regulation of *mir-93* and *mir-125A*, which are involved in invasion, migration and metastasis [33], [34], was observed in Cluster 4. Cluster 2 contained newly discovered *mir-1974*, a mitochondrialy-targeted miRNA [35] found to be decreased in adrenocortical carcinoma [36]. In addition to these specific miRNAs, other areas of smRNA dysregulation include transcripts that lead to alteration of transcription by modification of histone acetylation and methylation proteins. smRNA from several histone-associated proteins like Histone 1 complexes A-D as well as histone acetyltransferases (*MYST4*) and methylators (*MBD1*) were only found in TamR cells, and thus were not included in the clustering analysis in the ratio setting. However, such binary “on/off” expression suggests a strong role in mediating drug resistance.

In general, smRNA analysis of TamR and TamS breast cancer cells illuminated large alterations of snoRNA levels. This study provides support for the exploration of snoRNA in the cancer genome and drug resistance phenotype. Clustering analysis did not appear to cluster genes based on function, but analysis is restricted by the limited characterization of snoRNA and other ncRNA. As the field progresses, these snoRNA may be better categorized and the significance of the clusters may become apparent. Additional limitations of the smRNA analysis lie in the fact that some transcripts aligned to protein-coding exons of genes. While many of these genes may be subject to alternative splicing leading to the creation of smRNAs, the actual function of these smRNAs could be unrelated to the function of the gene. For this reason, we did not include analyses with these alignments. Overall, the existence of so many snoRNAs, miRNAs, and smRNA transcripts related to gene expression (histone modification, mitochondrial transcription, etc.) implicate the intricate regulation of a large set of gene expression changes in the development of Tam resistance.

### Gene ontology and clustering analysis of mRNA expression

To better understand the wide-range of altered mRNA transcripts and proteins in TamR breast cancer cells, we performed a gene ontology and pathway analysis of the differentially-expressed genes and clusters. Analysis was relegated to exon-significant mRNA transcripts since these can be verified as protein coding regions.

Using the absolute difference method, the majority of genes fell into one cluster, Cluster 2. Figure 3C shows that this cluster contains genes with low levels of expression and little absolute difference in gene expression. This is to be expected, as most transcripts are not highly expressed. Clusters 1 and 3 contain transcripts mostly from snoRNA regions, with varying levels of up- and down-regulation in TamR cells. While Cluster 2 contains snoRNA transcripts as well, it also includes miRNAs *mir1248*, *mir1291*, and *mir1978*, which are slightly up-regulated with moderate absolute expression levels in TamR cells. So far, these miRNAs have not been associated with any disease state. Transcripts for the non-protein coding *RMRP*, that was also found by smRNA analysis, clustered in this group as well. The assignment of *RMRP* and snoRNA to both smRNA and mRNA is unsurprising due to their intermediary sizes before processing ranging from 60–350 bp. Transcripts from the mRNA analysis designated as miRNA are probably the result of unprocessed transcripts or previously named miRNAs being assigned to areas of alternative splicing of unknown genes.

The clustering analysis gave a more substantial set of results for the mRNA transcripts using the ratio method that compares the relative difference of individual genes expression from TamS to TamR cells (Table 2). Of the three clustering groups, Cluster 1 contains all of the down-regulated genes in TamR cells. Analysis of the biological functions and pathways contained in Cluster 1 genes indicates a high level of modification of mitochondrial oxidative phosphorylation and gene expression regulation. Some of the ATP synthase genes are down-regulated by 2-fold, as are ribosomal proteins 60S proteins. Transcripts from splicesome genes *U2AF1* and *U2AF2* are decreased by 5- and 2-fold, respectively, in TamR cells. Expression of the transcription factor *JUNB*, which binds and represses AP-1, was decreased by 5.5-fold, which has previously been shown to be linked to increased cell cycle progression and lack of response to Tam [37]. In general, the down-regulation of Cluster 1 may be required for specific gene expression changes that allow Tam resistance to occur. Changes (up or down) in energy metabolism molecules have been observed previously with Tam treatment [38] and might be necessary for altered global gene expression.

**Table 2:** Functions of mRNA exon expression clusters in Tam-resistant cells.

	Category	Function	Molecules
**Cluster 1 (down-regulated)**			
	Mitochondria	ATP synthases	ATP: 5J2, 6V0E2
	Gene expression	Ribome 60S	RPL: 17, 27, 28, 35, 39, 41 , P0, P1
		Splicesome	U2AF: 1, 2
		Transcription factor	JUNB
		Histone-associated	HIST1H: 1C, 2AE, 2BD, 2BO, 3E, 4A, 4D
**Cluster 2 (moderately up-regulated)**			
	Gene expression	Histone-associated	HIST1H: 2AC, 2AM, 3F, 3J, 4H, HIST2H: 2AB, 2AC
		Histone-binding	HINT1
		Ribosome40S	RPS: 4X, 5, 6, 8, 21, 23, 24, 25,27
		Ribosome 60S	RPL: 3, 5,10A, 11, 13A, 23, 30, 36, 37, 38, P2
		Initiation factors	eIF: 2A, 3E, 3H, 3M, 4A1, 4G2, 5, 6
		Elongation factors	eEF1E1
		Proteosome	PSM: A1, A2, A4, A5, A7, B1, B2, C2, D6, D7, D10, D12, G3
	Mitochondria	NADH dehydrogenases	NDUF: A1, A4, A6, B2, C1, S3, S4
		ATP synthases	ATP: 1F1, 5A1, 5B, 5I, 5O, 6VOE1,6VOE1, 8B1,
		Cytochrome c	COX: 6C, 7A2L, 7B, 7C,16
		Mitochondrial ribosome proteins	MRPL: 16, 27, 32, 39, 47, 50, 53 MRPS: 7, 17, 21, 22, 23
	Cell cycle	Cyclins	CDK1, CDKN3, CCNB1, CCNC
		Retinoblastoma	RB1
**Cluster 3 (highly up-regulated)**			
	Mitochondria	ATP synthases	ATP: 5E, 6V1D, 6V1H
	Estrogen receptor	ESR1 pathway	CCNC, HRAS, MAPK1, NCOA, NRAS, NRIP1, PHB2, SRA1, TAF7
	Gene expression	HNF4a targets	ABD10, DPH5, NOP6, E2F
	Multi-drug resistance	Caveolins	CAV2

Cluster 2 exhibits a more moderate increase in gene expression of TamR cells, many of which are related to expression of transcripts and proteins. Pathway and function analysis shows this cluster to have the most diverse set of gene functions with alterations in mitochondria, transcription, translation, cell cycle, and ubiquitination. Transcription regulation is altered with a number of histone-associated genes that are up-regulated 2- to 3-fold as well as histone binding protein *HINT1*. Protein synthesis is affected on several levels. Ribosomal transcripts that code for proteins rather than rRNA were also increased such as 40S (RPSs: ribosomal protein S) small subunit and 60S (RPLs: ribosomal protein L) large subunit ribosomal proteins; interestingly, mitochondrial ribosomal proteins (MRPLs and MPRSs) were also increased. Another level of protein regulation was found with increases in translational machinery including up-regulation of initiation factors (eIFs), as well as increased elongation factor eEF1E1. Finally, post-translation modification is also up-regulated with an increase in proteosomal PSMs (proteosome/macropain) subunits.

The TamR breast cancer cells in Cluster 2 are also characterized by expression of cell cycle and mitochondrial energy metabolism genes. Molecules involved in the progression of cell cycle are moderately up-regulated in TamR cells. *Cyclin D1 kinase* (*CDK1*) and *Cyclin D3 kinase inhibitor* (*CDKN3*) are increased as are *Cyclin B* (*CCNB1*) and *Cyclin C* (*CCNC*). Master regulator *RB1* (*retinoblastoma 1*) is increased as well. We also found a 2-fold increase in various *E2F5* transcripts in TamR cells, as well as a decrease in *mir-16-2*, an E2F negative regulator miRNA which stops E2F1 control of proliferation [32]. The increase in E2F transcripts is probably partially due to the activation of HNF4a as they are known targets of the transcription factor. Multiple components of mitochondria are altered as well. In addition to the increase in mitochondrial specific ribosome proteins, NADH dehydrogenase subunits (NDUFs) are increased as are cytochrome c oxidases (COXs). Drug resistance has been previously linked to changes in cell cycle [39] and oxidative phosphorylation with a decreased use of glycolysis [39], [40].

Cluster 3 genes, which were highly up-regulated in TamR cells, also contained transcripts related to dysfunctional mitochondria and oxidative phosphorylation, in addition to those related to proliferation and drug resistance. Genes from the *ESR1* pathway were also increased including downstream proliferation activators and nuclear receptor regulators. These genes include *HRAS*, *MAPK1*, *NCOA*, *NRAS*, *NRIP1*, *SRA1*, and *TAF7*. Different ATP synthases were affected, increasing 4- to 7-fold in TamR cells. Interestingly, activation of transcription factor HNF4a (hepatocyte nuclear factor 4, alpha) genes was found with increases in targets without an increase in HNF4a mRNA. HNF4a has not previously been associated with breast cancer, but has been linked to types of ovarian and liver cancer [41] in addition to changing the expression of drug metabolism enzymes [42]. *Caveolin-2* (*CAV2*), which is involved in creating caveolae or invaginations of the cell membrane, was also increased ∼8-fold in Tam resistant cells. *CAV2* expression is associated with poor prognosis in breast cancer patients [43] and with multi-drug resistance in multiple cancer types [44]. Overall, Cluster 3 contains many of the traditional molecules associated with Tam resistance, including those related to *ESR1* along with multi-drug resistance molecules.

Taken as a whole, while all three clusters contain many of the same type of genes – mitochondrial and those related to gene expression – Cluster 2 stands apart with its inclusion of additional categories of modifications. Specific regulation of gene expression with histone modification, translation factors, and proteosome components is found exclusively in this cluster as are oxidative phosphorylation members relating to NADH dehydrogenases and cytochrome c. The designation of a variety of gene types to Cluster 2 is unsurprising since this cluster represents moderately altered genes, and most genes would be expected to only have moderate expression changes rather than dramatic ones.

### Comparison to traditional analysis methods and previous studies

A comparison of the ontology of genes found to be differentially-expressed between TamS and TamR cells validated the results of the NGS study. Numerous previous investigations have explored the mechanism of Tam resistance, from large microarray [13] and shRNA [16] screens to focused mechanism studies. These studies have linked pathways related to estrogen receptor to be of great importance to Tam resistance, which was confirmed by our study. Both the FET and two-standard deviation significance test showed that molecules related to ER-α (*ESR1*) pathways were found to be the most enriched genes as analyzed by GeneGo network and pathway analysis (Fig. 2C), such as *DDX5*, *MAPK*, and *NRIP* genes.

The other pathways implicated by the traditional two-standard deviation method are also in agreement with previous studies, including down-regulation of cell death/apoptosis molecules [45] and up-regulation of cell cycle regulators [46] and metabolic genes [47] (Fig. 2D). Tam resistant cell lines and tumors are known to have dysregulated cell cycle and apoptotic pathways in an attempt to survive long-term treatment and to overcome the cytostatic effects of Tam. One of our qRT-PCR genes confirmed to be up-regulated in TamR cells, the gene for Cyclin D1, has been shown to be increased in the plasma of breast cancer patients that have poor outcomes and are non-responsive to tamoxifen [48]. Although the FET analysis found many of the same metabolic and cell cycle regulators, apoptotic and cell death regulators were not among the most prominent molecules found by our new method, which may indicate that a variety of analytical methods should be used when exploring RNA-Seq data. While in agreement with the traditional ontology analysis, the expanded study, which uses the FET significance test and our model, gave a broader picture of the vast changes between these two cell lines. The ability of the TamR cells to change global expression of so many genes is highlighted by the amount of alteration at all levels of transcriptional and translational control including changes in epigenetic regulation, transcription factors, and post-translational regulation. smRNA is altered in both miRNA and snoRNA forms, emphasizing the complexity and dysregulation of smRNA in Tam resistant breast cancer. Previously known miRNAs were also implicated, with increases in *mir-21* expression which targets tumor suppressors [49], as well as up-regulation of *mir-93* and *mir-125A* [33], [34], which are both involved in the development of more aggressive phenotypes capable of migration and metastasis. These results together represent a global change in the way that genes are transcribed and expressed.

Additionally, along with traditional mechanisms of Tam resistance such as up-regulation of *ESR1* and other proliferative pathways, alterations in mitochondrial function occurred in TamR cells. This is not unexpected as new modes of energy metabolism would be necessary to have a global control of gene expression, and glycolysis may no longer provide adequate energy supplies. Altered oxidative phosphorylation has been linked previously to increased drug resistance [40].

Several large microarray studies on patient breast cancer tumors were previously reported which presented gene signatures associated with tamoxifen resistance and breast cancer recurrence. While these studies were limited by the fact that they did not explore smRNA expression, they did look at the prognostic value of gene expression signatures of tamoxifen resistant tumors. When the microarray gene signatures from Loi *et al* [50], Jansen *et al* [51], and Ma *et al* [52] were compared to our differentially expressed genes, relatively few genes overlapped. Out of the 81 transcripts reported as differentially expressed by Jansen et al., 73 could be matched confidently to our data. Of the 73 matched we observed 7 changed in expression. A similar 10% overlap in genes was found in our comparison with Loi et al where we confidently matched 173 out of 181 genes, while 17 out of these 173 changed expression in our dataset. We had no overlap with Ma *et al*. It should be noted that the gene signatures of these three previous microarray studies do not have a single gene that overlaps with one another, despite the studies using some of the same patient tumor datasets. In our case, this could be due to the difference in using a different technology. Microarrays measure one part of the gene, which are usually 3′ biased. With the sequencing approach, reads are measured across the gene. The differences could be attributed to the fact that these different methods are not analyzing the exact same thing. This is why prognostic values cannot be assigned to our gene results. While our current RNA-Seq data cannot be associated with a predictive gene signature for tamoxifen resistance as was produced by these previous microarray studies, the addition of patient samples to our analysis would make it possible to determine what genes are truly correlated with a response to tamoxifen in breast cancer patients. In general, there have been many challenges with the reproducibility of replication studies [53].

In fact, our own ontological analysis of the Loi *et al* [50], Jansen *et al* [51], and Ma *et al* [52] studies revealed that these predictive gene signatures shared little overlap in pathway and network analyses. Using both Ingenuity Pathway Analysis (IPA) and Roche GeneGo, we found a variety of different gene ontology terms for pathways and networks that were the most enriched for these different studies (Table S1), demonstrating the difficulty in studying the overlap between individual gene signature studies. A shared theme of cell cycle regulation, apoptosis, cytoskeleton rearrangement, DNA repair, immune response, small molecule biochemistry, and steroid receptor signaling were shared by the three studies and were confirmed in our own tamoxifen resistant dataset. The range of enriched pathways and networks found by these predictive gene expression signatures highlights the challenge in confirming significant results in tamoxifen resistance.

**Figure 5:**
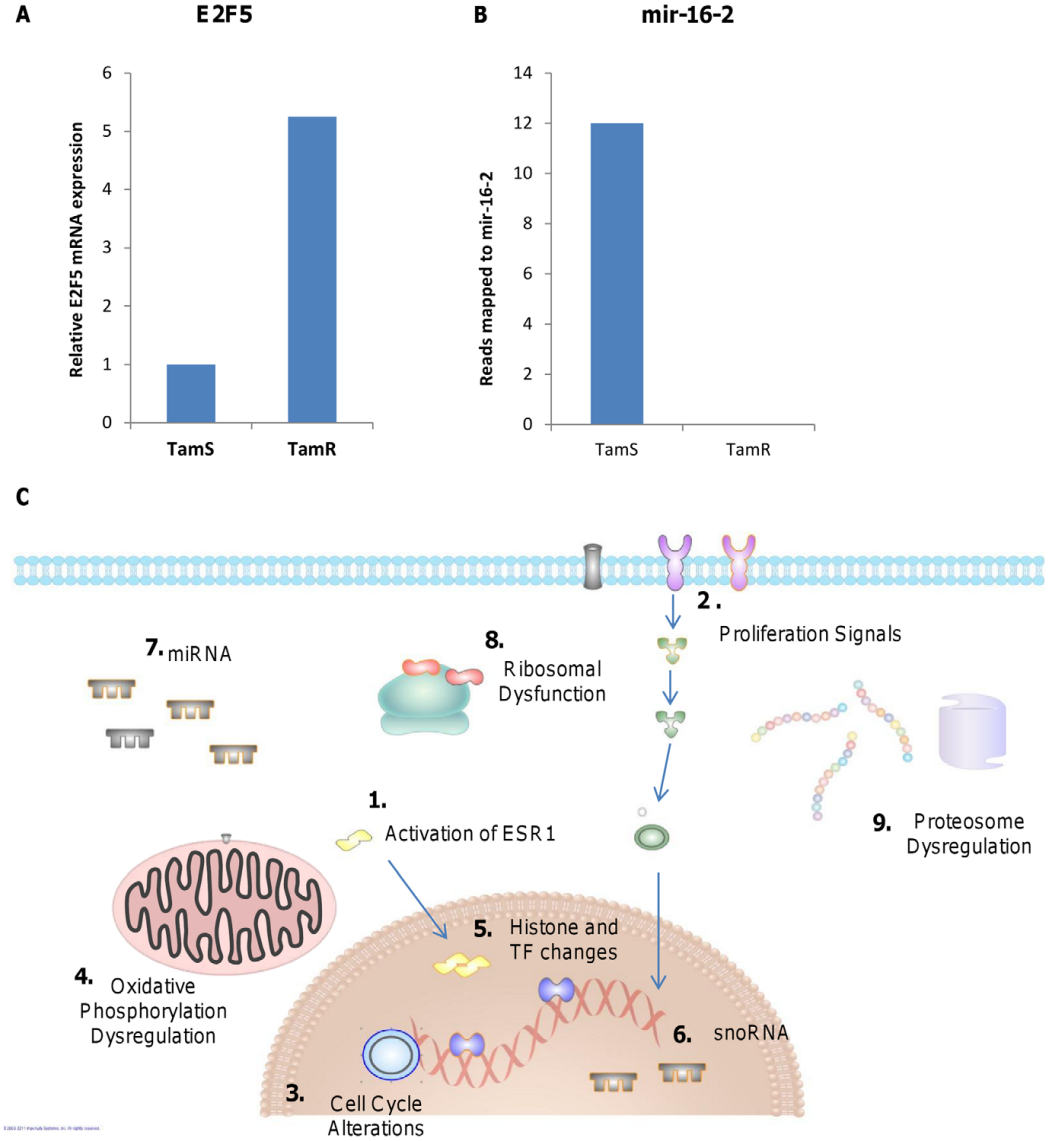
Dysregulation of pathways and processes involved in Tam resistance as revealed by NGS. Changes in E2F control of proliferation are in agreement with previous clinical sample studies with increases in E2F5 (**A**) and mir16-2 (**B**) expression in TamR cells. (**C**) Pathway analysis of clusters revealed several important areas of dysregulation in Tam resistance: traditional Tam resistant ESR1 (**1**) and proliferation (**2**) pathways are up-regulated in TamR cells, as are molecules involved in cell cycle progression (**3**). Oxidative phosphorylation is altered (**4**). Transcription was affected with modification of histone and transcription factor expression (**5**). Expression of transcripts was altered by the large number of smRNA molecules that were dysregulated, particularly in snoRNA (**6**) and miRNA (**7**) expression. Translation of proteins is affected in Tam resistance as well with up-regulation of ribosomal and translational machinery (**8**). Protein expression was also affected by an up-regulation of proteosomal proteins in TamR cells (**9**).

The diversity of molecules involved in Tam resistance was also established in a recent meta-analysis of three separate microarray studies [54]. The systematic study by Huang et al. examined the 275, 130, and 252 genes found in three public microarray data sets (GSE6532, GSE9195 and GSE9893), respectively, comparing Tam-sensitive and Tam-resistant breast cancer samples. While the authors found little overlap in the actual genes between datasets, they did find a general theme of cell cycle and proliferation transcription factor over-expression including an increase in activation of various E2F's in tamoxifen resistant cells in all three studies. In fact, E2F gene expression was the only common molecules between all three studies. They concluded that Tam-resistant cells were highly proliferative compared to their sensitive counterpart, a finding that is corroborated by our current study. Specifically, we also found an increase in *E2F5* transcripts in TamR cells (Fig. 5A), as well as a decrease in general E2F negative regulator *mir-16-*2 (Fig. 5B). Thus, while our study was performed in breast cancer cell lines, these findings support the validity of our method and its significance for clinical cases. Targeting of E2Fs may be a promising area for the development of adjuvant therapies that may sensitize breast cancer cells to Tam treatment.

## Conclusions

Our study highlights the ability of NGS to profile and characterize transcriptome changes in Tam-resistant breast cancer. RNA-Seq analysis of gene expression of Tam-sensitive and Tam-resistant breast cancer cells led to the identification of 1215 mRNA and 513 smRNA transcripts that were differentially-expressed. The sheer number of differentially-expressed genes demonstrates – quite effectively – that resistance to Tam is not through changes in an individual molecule or pathway, but is the result of global changes in gene expression (Figs. 4, 5).

RNA-Seq and NGS allow for an unbiased search for these pivotal transcriptome modifications. Regardless of these advantages, use of NGS has been limited by the lack of suitable analysis tools for the large amount of data generated. Our clustering method will add a means of determining the significance of similar levels of expression changes as found by NGS, which may, in due course, lead to the determination of molecules that induce these global or cluster changes. The ultimate goal of these studies was to identify molecules that could be exploited to modulate sensitivity of breast cancer cells to Tam. Although our study was focused on two breast cancer cell lines, these results will help with future studies with patient tumors that have the potential to identify new targets that are universally dysregulated in tamoxifen resistance. In particular, as total smRNA on an NGS platform has not previously been used to characterize Tam resistant tumors, further research in this specific area of study would help to identify which smRNAs are universally dysregulated. In light of the large number of gene expression changes found, it is conceivable that some alterations are the driving changes leading to Tam resistance

Our findings reveal three areas that are modified on multiple levels in our Tam-resistant cells. First, proliferation signaling is modified with changes in cell cycle control and ESR1 down-stream genes that permit unregulated proliferation of the Tam-resistant cells (Fig. 5C1, 2, 3). Second, mitochondria and oxidative phosphorylation are affected (Fig. 5C4). Several different types of units in the electron transport chain are altered that may permit new and more efficient means of energy production for the breast cancer cells. Finally, this study indicates that gene expression regulation is dramatically altered from changes in transcriptional control to adjustments in post-translational modifications and protein degradation (Fig 5C5, 6, 7, 8, 9). Within this area, we find that snoRNA could play a major role in Tam resistance. Independently, each of these areas could be investigated for therapeutic targets, and further exploration of the changes in snoRNA may lead to new diagnostic tests for Tam resistance. Together, our results exemplify the need for personalized medicine as the large number of genetic changes in Tam resistance can be overwhelming, but patterns of dysregulation may emerge as a patient's own genetic signature is compared to samples of known resistant phenotypes. Using NGS and clustering methods, therapies may be developed that target proteins or genes that are found to regulate these global changes, sensitizing more breast cancers to the anti-proliferative effects of Tam.

## Materials and Methods

### Cell lines

Parental MCF-7 cells were grown in DMEM (Hyclone) supplemented with 5% fetal bovine serum (FBS) (Hyclone), 100 units/mL penicillin, and 100 µg/mL streptomycin. The MTR-3 line (MCF-7 Tamoxifen-Resistant-3) was derived from the parental MCF-7 cells by continuously culturing the cells in the presence of 1 µM Tam (Sigma Aldrich) in phenol red-free DMEM (Hyclone) supplemented with 5% charcoal/dextran-stripped fetal bovine serum (CSS) (Hyclone) and antibiotics. Estrogen independent cells (MCF-7-E2) were derived from parental MCF-7 cells grown in phenol red-free DMEM (Hyclone) supplemented with 5% CSS (Hyclone) and antibiotics. Cells were maintained at 37°C in a humidified atmosphere containing 5% CO_2_/95% air.

### RNA preparation

Total RNA was prepared with TRIZOL Reagent (Invitrogen) from MCF-7 and MTR-3 cells grown under preferred culture conditions as described above. RNA was extracted and isolated as recommended by the manufacturer. Sample integrity was verified with Nanodrop 1000 and Agilent Bioanalyzer 2100.

### Library preparation for SOLiD™ NGS sequencing

Library preparation for both whole-transcriptome (WT) sequencing and small RNA (smRNA) sequencing was performed using the smRNA expression Kit (Applied Biosystems Inc,) based on SOLiD WT and smRNA sequencing protocols provided by ABI. rRNAs were depleted from total RNA using the Ribominus Eukaryotic Kit (Invitrogen). rRNA-depleted total RNA (0.5–1.0 µg) was fragmented by RNase III. The fragmented rRNA-depleted total RNA were hybridized and ligated with adaptors, followed by reverse transcription. The cDNA were size-selected in 100∼200 nts, amplified (12–15 cycles), and re-size-selected as recommended by ABI. The purified PCR products were size-selected in the range of 150∼250 bp, containing 50–150 bp cDNA inserts (quantitated and qualified by Agilent Bioanalyzer 2100) for WT and 108–135 bp PCR products containing 18–40 nt of smRNA inserts to make libraries.

### Sequencing

The individual prepared libraries were quantitated as templates for emulsion PCR; the template molecules were attached to beads, enriched for adaptor P2, and immobilized to the slide according to the ABI SOLiD emulsion. The sequencing runs were performed on a SOLiD v 3.5 for both WT-seq and small RNA-Seq. The number of P2 positive template beads (equal to the number of transcripts) deposited on the sample slide were 71,250,509 and 69,005,180 of 50 nt length for WT sequencing, and P2 positive beads for small RNA sequencing were 35,686,597 and 35,176,389 of 35 nt length for smRNA sequencing, of TamS and TamR cell lines, respectively

### NGS mapping and expression

Fifty bp and thirty-five bp reads (for WT and smRNA, respecitively) were assessed for quality and mapped to the reference human genome (hg18) by the software Maq: Mapping and Assembly of Qualities. Whole transcriptomes for the two cell lines were constructed and compared for their gene expression. One hundred and forty million total reads were produced by sequencing, and ∼50% of them mapped to the genome after initial quality control measures. Applied Biosystems WT and smRNA Analysis Pipelines were used to confirm results and score expression. Histogram analysis of the log2(# of case reads/# of control reads) provided gene candidates that were differently expressed between the tamoxifen sensitive and resistant cells with a 1.7 fold criterion for the traditional model.

### qRT-PCR validation

Potential gene candidates were validated using TaqMan Gene Expression assays. cDNA was made from previously harvested total RNA of MCF-7, MCF-7 estrogen-independent cells, and MTR-3 cells (Roche). The products were tested for purity using spectrophotometry (Aligent Nanodrop). RT-PCR was performed using TaqMan Gene Expression Assays (Applied Biosystems) on a Statagene Mx3005P (Aligent Technologies). GAPDH was used to normalize samples for comparison.

### Statistical models

We implemented a novel statistical model for identifying the patterns and differences in smRNA and mRNA expression in TamS and TamR cells. Consider *m* genes are detected in both TamS and TamR cells. Because of their functional similarities and differences, these genes can be clustered into different groups. Let (*y*_1*i*_,*y*_2*i*_) denote the expression data for gene *i* from these two cell lines, respectively. We can describe the differential expression of gene *i* using the absolute difference (i.e., *y_i_* = *y*_1*i*_−*y*_2*i*_) or ratio (*z_i_* = *y*_2*i*_/*y*_1*i*_) of the gene's expression between the two cell types. Genes will be clustered into different groups based on the differences and ratios between individual genes in the TamR and Tams S cell lines using a mixture-based likelihood model:
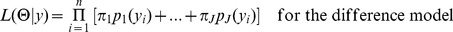
(1)

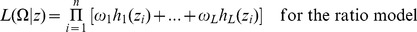
(2)where 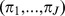 and 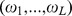 are a set of proportions that each correspond to a different gene group under the difference and ratio model, respectively; *p_j_*(*y_i_*) and *h_l_*(*z_i_*) are the discrete probability distributions of differential expression for group *j* (*j* = 1, …, *J*) for the difference model and group *l* (*l* = 1, …, *L*) for the ratio model. The expression reads of genes in each cell type are thought to obey a Poisson distribution (16), thus the distribution of the read differences and ratios between the two cell types is modeled by specific functions.

The EM (expectation maximization) algorithm can be conveniently used to estimate the means of gene expression in TamS (*μ_Sj_*) and TamR cells (*μ_Rj_*) for group *j* under the difference model. Similarly, the means of gene expression in TamS (*μ_Sl_*) and TamR cells (*μ_Rl_*) for group *l* can also be estimated. The optimal number of clusters is determined by a model selection criterion, such as commonly used Akaike information criterion (AIC) [28] or Bayesian information criterion (BIC) [29]. In this article, both the AIC and BIC values under different numbers of clusters were calculated to be the same; thus, only the AIC values were reported. The optimal number of clusters corresponds to the minimum AIC value,. After this is determined, our model allows the following biologically meaningful tests.

Test 1: For a given cluster group, do TamS and TamR cells differ? This can be done by testing:
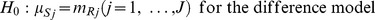


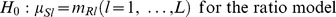



If the *H*_0_ is rejected, this group of genes is expressed differently between TamS and TamR cells, indicating that they may be involved in drug resistance and can be viewed as a biomarker of drug response;

Test 2: For a pair of genes, do they interact with each other to determine drug resistance? This can be done by testing:;
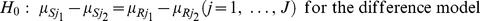


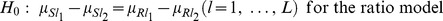



A rejection means that these two groups of genes have significant interaction effects on drug response.

### Expression Analysis

Gene network and pathway analyses were conducted using the Ingenuity Pathway Analysis (IPA, Ingenuity® Systems) and GeneGO (Thomson Reuters) software. Functional analysis of the resistant cell lines was performed using IPA with a 1.7-fold change criteria and a P value of <0.01.

## Supporting Information

Table S1 **Difference in Gene Ontology (GO) terms for gene signature of significant Tam resistance studies.**
(DOCX)
