# Supplementary material for: Differential Gene Expression in Tamoxifen-Resistant Breast Cancer Cells Revealed by a New Analytical Model of RNA-Seq Data
Source: PLoS One. 2012 Jul 23;7(7):e41333. doi: 10.1371/journal.pone.0041333 (PMC3402532; doi:10.1371/journal.pone.0041333)
Supplement: Table S1 — Difference in Gene Ontology (GO) terms for gene signature of significant Tam resistance studies. (DOCX) [file pone.0041333.s001.docx]

**Table S1: Difference in Gene Ontology (GO) terms for gene signature of significant Tam resistance studies.**

|  | **Our Genes** | **Loi *et al*** | **Jansen *et al*** | **Ma *et al*** |
| --- | --- | --- | --- | --- |
| **GeneGo Pathway** | Oxidative Phosphorylation | Cell Cycle | Glycolysis | Immune System |
|  | Ubiquinone | Metabolism | Cell Adhesion | Cell Signaling |
|  | Cytoskeleton | Immune System | Cytoskeleton | Cell Adhesion |
|  | Translation | Reproduction | Proteolysis | Apoptosis |
|  | Apoptosis | MAPK | Immune System |  |
|  |  |  |  |  |
| **IPA Functions** | Protein Synthesis | Cell Cycle | Development | Cell Movement |
|  | Post-Trans Mod | Growth/Prolif | Small Molecule Biochemistry | Cell Compromise |
|  | Growth/Proliferation | Cell Death | Cell Morph | Cell-To-Cell |
|  | Metabolism | Cell Assembly | Cell-To-Cell | Antigen |
|  | Small Molecule Biochemistry | DNA Repair | Lipid Metabolism | Cell Assembly |
|  |  |  |  |  |
| **GeneGo Networks** | Estrogen Receptor | Cell Cycle | Cell Adhesion | Proteoloysis |
|  | Translation | Cytoskeleton | Wnt Signaling | Nuclear Receptor Signaling |
|  | Proteolysis | Steroid Synthesis | Proteolysis |  |
|  | Immune System | DNA Damage | Apoptosis |  |
|  | Cell Cycle | Proteolysis | Development |  |
|  |  |  |  |  |
| **IPA Networks** | Protein Synthesis | Cell Cycle | Development | Inflammation |
|  | Small Molecule Biochemistry | DNA Repair | Developmental Disorders | Small Molecule Biochemistry |
|  | DNA Repair | Antigen | Small Molecule Biochemistry | Cell Assembly |
|  | Gene Expression | Cell Growth | Cell-To-Cell |  |
|  | Cell Cycle | Small Mol | Cell Develop |  |
